# Angiotensin II type 1 receptor blocker losartan attenuates locomotor, anxiety-like behavior, and passive avoidance learning deficits in a sub-chronic stress model

**DOI:** 10.22038/IJBMS.2018.27113.6632

**Published:** 2018-08

**Authors:** Hoda Ranjbar, Iraj Aghaei, Mahmood Moosazadeh, Mohammad Shabani

**Affiliations:** 1 Intracellular Recording Lab, Kerman Neuroscience Research Center, Neuropharmacology Institute, Kerman University of Medical Sciences, Kerman, Iran; 2 Social Determinants of Health Research Center, Guilan University of Medical Sciences, Rasht, Iran; 3 Health Science Research Center, Addiction Institute, Mazandaran University of Medical Sciences, Sari, Iran

**Keywords:** Acetylsalicylic acid, Antioxidants, Epididymis, Melatonin, Sperm, Testosterone

## Abstract

**Objective(s):**

Stress alters sensory and cognitive function in humans and animals. Angiotensin (AT) receptors have demonstrated well-established interactions in sets of physiological phenomena. AT1 receptors can play a part in stress-induced activation of hypothalamic-pituitary-adrenal (HPA) axis; besides angiotensinergic neurotransmission plays a pivotal role in stress-evoked physiological responses. AT1 receptors are also involved in nociception and memory. The objective of the current study was to evaluate the effects of losartan as an AT1R antagonist in locomotor activity, nociception and memory impairments induced by sub-chronic swim stress.

**Materials and Methods:**

A two-session forced swimming stress protocol was administered to the rats. Pretreatment with losartan (10 mg/kg, IP) or saline was made before each swimming session. Locomotor activity, anxiety-like behavior, nociception, and passive avoidance learning were evaluated 24 hr after last swim stress session.

**Results:**

Swim stress induced increased anxiety-like behavior in the open field test, which pretreatment with losartan did counterbalance. Increased thermal threshold was observed in the nociceptive measurement after swim stress. Pretreatment with losartan attenuated the increased threshold and also inhibited a decreased step-through latency that was observed in the memory paradigm after swim stress.

**Conclusion:**

The results of this study indicate that sub-chronic swim stress impairs passive avoidance learning, anxiety-like behaviors, and nociception; and AT1 receptor seems to have a modulatory role in these alterations. However, further studies are suggested to examine the protective effect of AT1R inhibitors on stress-induced impairments in sensory and cognitive function.

## Introduction

Stress is indicated as a risk factor for mental and psychological disorders, including depression and anxiety disorders (1, 2), which leads to disturbance of brain homeostasis (3). Stress is associated with defensive behaviors that are involved in emotional states of anxiety (4). Previous studies reported that stress has a considerable impact on impairing exploration and locomotor activities (5). 

However, responding to stress depends on various factors including, ability to adapt to stress, which is related to sex, age, genetics, duration, and environmental influences (6). Therefore, it should be noted that depending on the type of stress different results are expected. Forced swim stress is one of the effective tests for the interpretation of anxiety and depression-like behavior. Hence, in the present work, it was used to cause psychological disorders (7).

In terms of detrimental effects of stress, anxious conditions have different complex effects (e.g. facilitating, impairing, and neutral) on memory (6). Although this demonstrates the multifaceted effects of stress on memory processing, numerous studies have emphasized the cognitive deficits and emotional processes related to memory caused by stress (8). Stress-induced memory deficits have raised many clinical efforts to develop medications, which appear to have some benefit on cognitive functions. Losartan is a widely used antihypertensive drug in patients, interestingly, recent evidence considered it as an effective drug in the treatment of memory defects (9). Thus, we decided to examine these claims by using passive avoidance test to evaluate memory processes and losartan impacts on it. 

In the recent decades, scientists showed interest in understanding the interactions between stress and pain. These data suggest that the nature, duration, and intensity of the stressor are key factors of the effects of stress on pain (10, 11). Pain is a very subjective phenomenon, difficult to quantify but at the same time can be problematic if left untreated (12).

It is important to note that the stimulatory effect of psychological stress induces physiological activity on pain sensitivity. Indeed, chronic exposure to physical or psychological stressors results in stress-induced hyperalgesia (SIH) (10).

The changes in pain sensitivity seem to be modulated by the types of stimuli used for inducing stress. Several theoretical frameworks have been proposed (13). In our study, we used forced swim stress (FSS). Hyperalgesia in rats exposed to repeated swim-stress has been demonstrated (14), which confirms FSS as a useful model for studying SIH. In addition, recent studies have shown that the administration of losartan plays a role in modulation of the pain system (15, 16). Thus, this study was undertaken to evaluate the effects of losartan on pain perception in rats after receiving FSS.

In general, the present study was designed to investigate the advantages and drawbacks of losartan administration on stress symptoms, particularly its effects on anxiety behavior, memory processes, and pain.

## Materials and Methods

***Experimental animal*** *s*

The experiments were performed on 64 male Wistar rats (180–220 g), which were obtained from the laboratory animals of the Neuroscience Research Center of Kerman Medical University and the study protocol was approved by The Animal Ethics Committee of this institution [code: EC/KNRC/89-169]. Rats were housed under standard laboratory conditions with a 12 hr light/dark cycle (lights on at 07:00, off at 19:00) at a constant temperature (22±2 °C). Food and water were available *ad libitum*, except during the stress condition. After the last session of stress, the behavioral experiments were carried out in all groups. Animals were randomly assigned to two groups (sham and stress) and four subgroups (pain and cognition tasks with losartan/saline) in each group (n =8) as follows: 

***A, Sham swim (SS):***

**1 and 2.** Normal saline + sham (Ns +Sham): (group1 for nociception tasks including hot plate and tail flick tests, group 2 for cognition tasks including passive avoidance test (PA) and open field test (OFT))

**3 and 4.** Losartan +Sham: (group 3 for nociception tasks and group 4 for cognition tasks)

***B, Forced swim (FS):***

**5 and 6: **Normal saline + stress (Ns +Stress): (group 5 for nociception tasks and group 6 for cognition tasks)

**7 and 8:** Losartan +stress: (group 7 for nociception tasks and group 8 for cognition tasks)

Saline group’s rats were pre-treated with saline (0.5ml, IP) and Losartan groups received losartan (10 mg/kg, IP) 30 min before sham conditions or swimming session.

***Experimental procedures***

Stress paradigms

*Forced swim stress (FSS)*

The FSS test was developed as a model for screening losartan effects on stress. Rats were placed in the testing room for at least one hour before the start of the experiment. The FSS paradigm involved placing rats in a cylinder 50 cm in diameter and 50 cm in height, filled with 20 °±1 °C water to a height of 20 cm, for a period of 10 min on the first day, and 20 min duration on the subsequent two days (7). 

Animals with sham swim stress were placed in the same cylinder that contained only 2–4 cm of water at 20 °C. After swimming sessions, each rat was carefully dried with a new towel. At the end animals were transferred to the home cage (17).

Behavioral apparatus and methods

*Open field test*

This test was performed in a soundproof, air-conditioned chamber under dim light. Testing was carried out in a square Plexiglas open field (90 × 90 × 30 [H] cm) (18). Squares (n=12) adjacent to the walls were considered as the peripheral zone, whereas the remaining squares (n = 4) were considered as the central zone. The open field box was cleaned with alcohol thoroughly between sessions to assess total locomotor behavior and anxiety-like responses. Rats were placed in the center of the open field box. Total movement was tracked for 5 min and analyzed for evaluation of anxiety behavior such as the time spent in the center of the open field (19), total distance moved, velocity, grooming, and total locomotor activity (20). Less time spent in the central area reflects decreased exploratory and increased anxiety behaviors. Also, total distance traveled was used as a measurement of locomotor activity (21).

*Hot plate*

The animals in each group were placed one by one on the hot plate for evaluation of pain sensitivity (with 19 cm diameter and 30 cm height), which was surrounded by a transparent Plexiglas chamber with an open top, the reaction time was noted at 30, 60, and 90 min intervals. Reaction time is the time to respond with either a hind paw lick or hind paw flick. This behavior was considered the end point of the pain response. Immediately after a response rats were removed from the hot plate. Animals were also removed if they did not respond after 30 sec, to prevent tissue injury. The surface of the hot plate was heated to a constant temperature of 52±2 ^°^C (22, 23).

*Tail flick*

Every time before behavioral testing, rats were habituated to the experimenter and the room in which the behavioral experiments took place for at least half an hour. 

The tail flick test, as an acute model of pain, assesses the antinociceptive effect of drugs by measuring the latency time (24). Latency time is the time from the onset of heat exposure to the withdrawal of the tail. Tail flick radiant heat (adjusted to yield baseline latencies of 2–4 sec) was applied to tail at 5–8 cm from the tip. The cut-off point as tail response sufficient to interrupt the tissue damage was established at 10 sec. The animals showing baseline latency times of less than 2 or more than 4 sec were excluded from the study. The latency times were determined in 15 min intervals. Antinociception was quantified as tail flick latency time (25).

*Passive avoidance test*

A shuttle-box apparatus was used for passive avoidance test. The apparatus consisted of one lighted chamber and one dark chamber with grid door. Electrical shocks were transferred by a separated stimulator to the grid floor of the shuttle box. This test was performed for each rat for two consecutive days. In the first day, each rat was put in the device to habituate. After habituation, an acquisition trial was performed in which animals were initially placed in the lighted compartment and the door between the two compartments was opened. The initial latency for a rat to enter the dark compartment was measured. After entering the dark compartment, the door was closed and an electric foot-shock (0.5 mA, 50 Hz, 2 sec once) was delivered through the stainless steel rods. All animals examined, entered the dark compartment within 60 sec as cutoff latency in the training session, and received a foot-shock. Step-through latency (STL) for animals was recorded on the second day using the same paradigm, but without foot-shock (20, 26). 

Corticosterone level

One day after swimming sessions (8:00 am), animals were anesthetized using carbon dioxide (CO_2_) and were killed by decapitation. Trunk blood was collected and centrifuged at 2600 rpm for 20 min. The serum was refrigerated at −80 ^°^C until the day of analysis. Samples were then analyzed blindly by an ELISA kit used specifically for rats.

Drugs and treatments 

Animals were randomly divided into 8 groups, each group receiving one of the following treatments: normal saline (0.5 cc) [FS (pain and PA tasks subgroups) and SS (pain and PA tasks subgroups)], losartan (Ang II receptor blockade, 10 mg/kg, IP) [FS (pain and PA tasks subgroups) and SS (pain and PA tasks subgroups)] (27). 

***Data analysis***

All data are reported as the mean ± SEM. The data of various groups (between groups) were compared using two way ANOVA followed by Tukey’s test for multiple comparisons. Two-way ANOVA was performed to compare a significant interaction of stress (sham or stress condition) × treatment (losartan or saline) for locomotor activity in rotarod, pain threshold in tail flick and hot plate, and procedure of learning in passive avoidance. A *P-value* of less than 0.05 was considered statistically significant. 

## Results

***Effect of forced swim stress and losartan on locomotor and anxiety-related behaviors***

**Figure 1:**
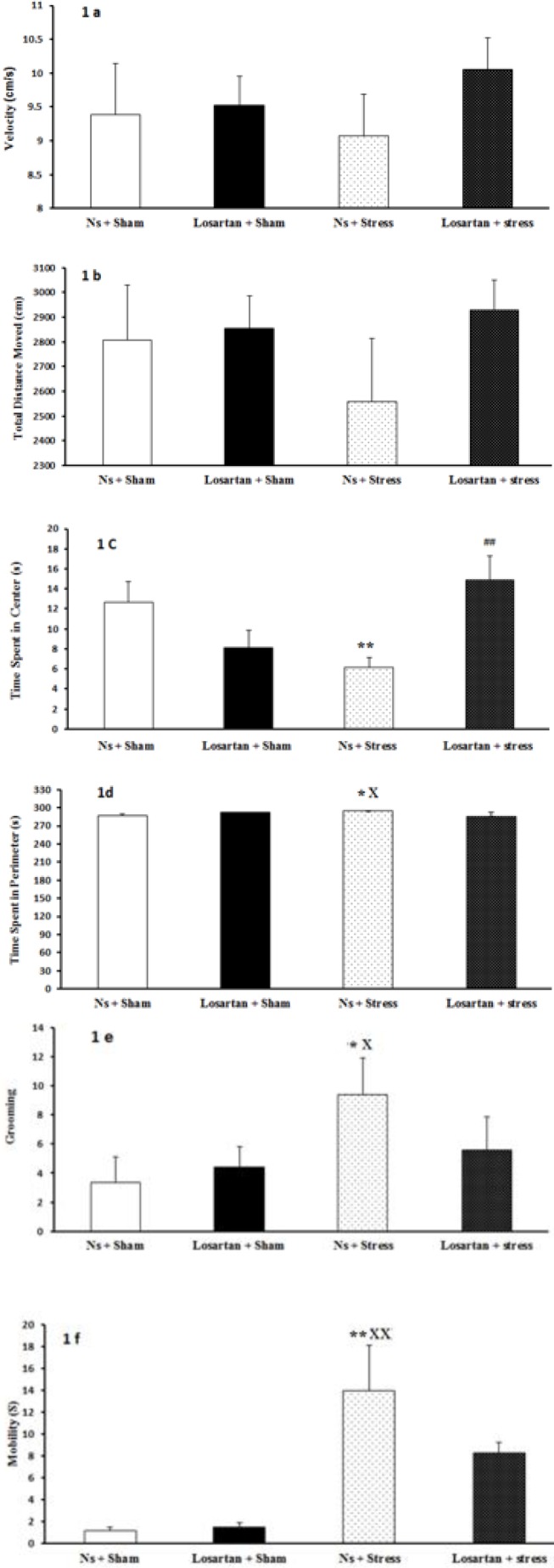
Effect of stress and losartan on A) velocity, B) total distance moved, C) time spent in the center, D) time spent in perimeter area, E) grooming, and F) mobility in open field test. Results are expressed as mean±SEM (ANOVA test, for comparisons between groups; *P<0.05, **P<0.01 when compared to Ns+Sham group; x*P*<0.05, xx*P*<0.01 when compared to Losartan+Sham group; ##*P*<0.01 when compared to Ns +Stress group)

The open-field test was conducted to examine locomotor activity and anxiety-related behaviors. In this test, stress altered total locomotor activity (Figure 1). Data showed no significant difference in velocity (F (3, 28) =0.49, *P>*0.05; Figure 1a) and total distance moved (F (3, 28) =0.68, *P>*0.05; Figure 1b) in all groups compared to the control group. A two way ANOVA revealed that stress significantly altered time spent in the center (F (3, 28) =3.62, *P<*0.01; Figure 1c). Forced swim rats stayed less in the center in comparison to sham rats (Ns +Sham and Losartan +Sham groups). Losartan + stress group rats stayed more in the center of the open field compared to forced swim rats, indicating losartan decreased anxiety and stress behavior by enhancement of tendency to spend more time in the center area. The time spent in perimeter area in Ns +Stress group, were significantly higher than those in the Ns +Sham and Losartan +Sham groups (F (3, 28) =2.8*, P<*0.05; Figure 1d). 

Stress had significant alternation in grooming (F (3, 28) =1.26, *P<*0.05; Figure 1e), which increased aggressive grooming compared to the sham groups. In line with our findings, some authors proposed that grooming behavior is linked with an anxiolytic state in rodents and partially explains anxiety states. 

The mobility of the Ns +Stress group was increased when compared to Ns +Sham and Losartan +Sham groups (*P<*0.01 in both cases). No differences, however, were found in other groups (F (3, 28) =3.38 *P>*0.05; Figure 1f). 

***Effect of swim stress and pretreatment with losartan on passive avoidance learning***

**Figure 2:**
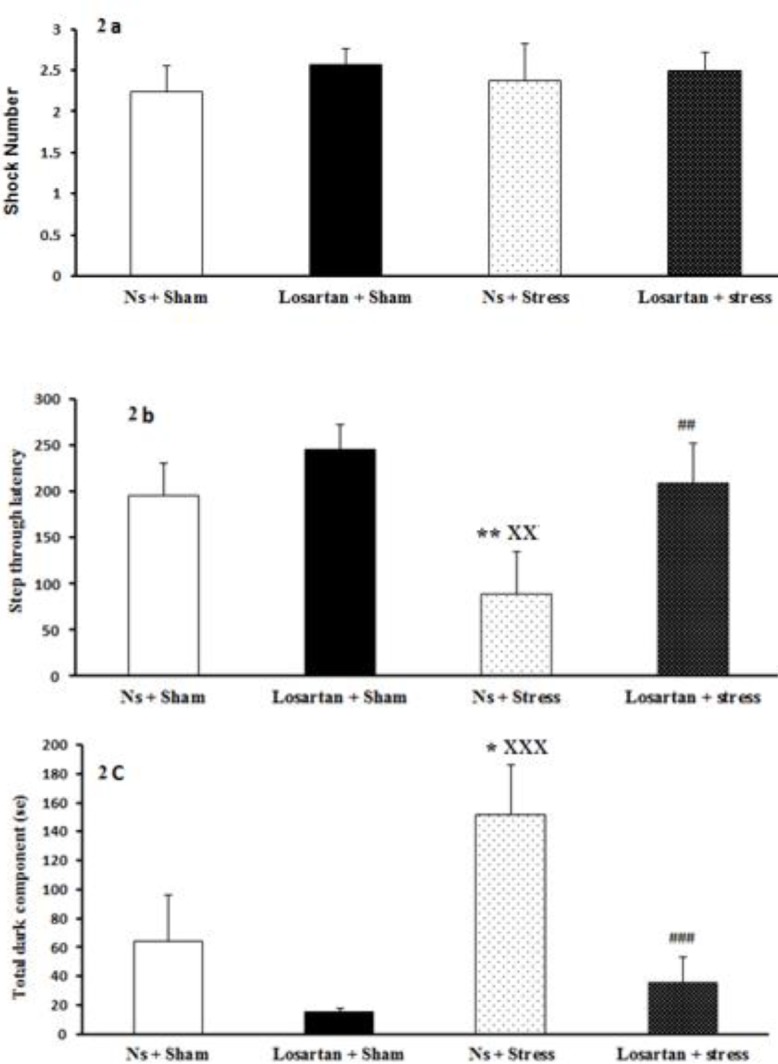
Effect of stress and losartan in passive avoidance test. A) shock number, B) step through latency, C) total dark component. Results are expressed as mean±SEM (ANOVA test, Tukey’s test for comparisons between groups; *P<0.05, **P<0.01 when compared to Ns+Sham group; xx*P*<0.01, xxx*P*<0.001 when compared to Losartan+Sham group; ## *P*<0.01, ###*P*<0.001 when compared to Ns +Stress group)

FSS and pretreatment with losartan did not significantly change the number of shocks compared to the sham swim group (F (3, 28) =0.18, *P>*0.05; Figure 2a). Step through latency was recorded for all groups. Interestingly, latency to enter the dark compartment was decreased in the Ns + stress group compared with both Ns + sham and Losartan + sham groups (F (3, 28) =3.12, *P<*0.05, *P<*0.001, respectively; Figure 2b). Whereas, Losartan + stress rats significantly (*P<*0.01) showed less tendency toward the dark chamber compared to the Ns + stress group. It may indicate the positive effects of losartan on lowering the harmful impact of stress on memory.

As expected the total dark component in the Ns + stress group increased compared with their own sham group (*P<*0.05). Meanwhile, Losartan + stress group showed a reduction in the time spent in the dark side when compared to Ns + stress group (F (3, 28) =6.2, *P<*0.001; Figure 2c). 

***Effect of swim stress and pretreatment with losartan on thermal pain thresholds***

**Figure 3:**
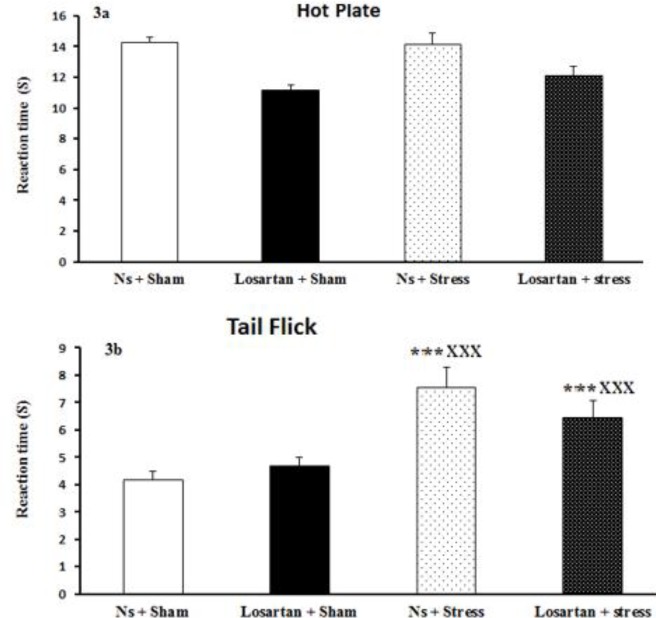
Effect of stress and losartan on pain in A) hot plate and B) tail flick tests. Results are expressed as mean±SEM (ANOVA test for comparisons between groups; ***P<0.001 when compared to Ns +Sham group; xxx*P*<0.001 when compared to Losartan+Sham group)

Hot plate test was performed to evaluate the pain sensitivity before and after the induction of stress. However, no significant differences were detected in any experimental group (Figure 3a). Thermal pain threshold was significantly increased in forced swim groups in the tail flick test compared to sham groups (F (3, 28) =8.9, *P<*0.001; Figure 3b), while no differences between other groups were observed and losartan could not compensate this effect of swim stress. 

***The effect of stress and losartan on corticosterone level***

Plasma corticosterone levels in none of the non-FSS groups showed any changes during the experimental period. Two-way ANOVA showed a significant higher plasma corticosterone level in the FSS treated groups (Table 1). However, there were no significant differences by losartan supplementation. Exposing to losartan did not change the pattern of the corticosterone response to FSS in the male rats compared to the NS +Stress rats, which were exposed to FSS during the experiments. The two-way ANOVA analysis showed no significant interaction or significant main effect of losartan on serum concentration of corticosterone.

## Discussion

The concept of this study was based on the different impacts of losartan on anxiety responses, cognitive processes, and pain. While this matter has already been discussed from various points of view, interpretation of different aspects of this drug’s action is difficult if not impossible. Therefore, we highlight our findings on this controversial notion in the following section.

**Table 1:** Plasma corticosterone level

	Plasma corticosterone level (pg/ml)
Ns +Sham	89.56± 6.09
Losartan + Sham	95.33±11.34
Ns +Stress	118.14±7.19^*^^x^
Losartan + Stress	106.28±13.08

Changes in plasma corticosterone levels after FSS and losartan administration. Data are presented as mean±SEM. Statistical analysis was performed by two-way ANOVA.

* *P*<0.05, when compared to Ns +Sham group;

x *P*<0.05, when compared to Losartan+Sham group

In the present study, we examined the effect of losartan on FSS and also the changes that losartan caused in the motor and anxiety-like behavior. Anxiety states were assessed with open field test, which is a standard behavioral model in rodents (28). Our finding indicated that losartan is involved in decreasing anxious behaviors and prevented stress responses that are caused by FSS. This test gives a valuable measure in the understanding of anxious behavior and locomotor activity. Center/ perimeter areas express an alteration of preference, from the less dangerous or exposed position (perimeter areas) to the higher risk possibility (center areas). This parameter is a manifestation of the anxiolytic effect of stressed rats. An increase in central areas duration is a parameter classically linked to locomotor activity (29). The reduction found in the time spent in the center in Ns + stress compared to Ns + sham strongly indicates the increasing anxiety in this group. In contrast, Losartan + stress group increased central time compared to Ns + stress group. This is in line with previous findings, showing that losartan reduces the anxious behaviors caused by stress (30). In rodents, an increase in fear response has been associated with impairment in exploratory and locomotor activities through heightening anxiety-like behavior such as increase in the frequency of defecation, and higher grooming and rearing frequency (29).

Hyperactivity of Ns + stress animals was shown by more tendencies towards perimeter areas of the open field compared to Ns + sham. Interestingly, mobility alternations were consistent with the previous parameter and showed motor hyperactivity in rats which normal saline and stress induced. Meanwhile, losartan did not affect total distance moved in sham and losartan groups compared to the saline-injected group. The velocity followed the same pattern and was not affected in any of the experimental conditions. Another parameter was the grooming behavior, characterized as a response associated with the stressful situation. Therefore, the animals under stress would spend more time in grooming than the animals in the control condition (31). This could explain our findings with stress that significantly increased the grooming behavior. However, it seems losartan injection had no significant effect on the grooming behavior induced by stress. It was suggested that stress induces activities that represent a diminished motivation to interact with the environment, which would explain the decreased exploratory behavior after FSS in our study. This is also supported by the finding that stressed rats increased grooming behavior, which is considered a behavioral response that follows alterations provoked by anxiogenic stimuli (4). However, the results of the present study showed that the corticosterone levels after the FSS assay did change and this finding was in line with a study that was conducted by Takeuchi *et al *(32). The acute stress-induced glucocorticoid increase is usually a beneficial response that helps the body avoid injury. Although the plasma corticosterone level on the day after stress session tended to decrease in the Losartan + Stress group, two-way ANOVA didn’t show any significant changes by Angiotensin II type 1 receptor blocker. 

The losartan treatment of stress induced in the present study can be explained by previous data showing that chronic blockade of the AT 1 receptor within amygdala improved anxiety responses (33). Moreover, recent clinical uses give additional support to the idea that AT1 receptor inhibitors could potentially be used as anxiolytic drugs. Thus, Administration of losartan which is a selective AT1 receptor antagonist might reduce motor hyperactivity and anxiogenic behavior in stressed rats via inhibition of AT 1 receptor in the amygdala (29).

Increasing evidence suggests that losartan plays a role not only in the reduction of anxiety behavior but also in learning and memory (31). In agreement with these reports, the present work showed that injection of losartan significantly improved learning and memory, examined using the passive and active avoidance tests. In the shuttle box test, losartan significantly increased the step-through latency during the retention test (memory), while the injection of normal saline did not show a significant effect (9). 

The present results showed that losartan improved memory function. It can be concluded that losartan has a positive effect not only on blood pressure but also on memory function. The possible mechanism would be the involvement of brain angiotensin II (AII) in cognitive processes, including learning and memory (34). The hippocampus is a key brain structure in memory formation. Previous studies indicated that administered losartan (an antagonist of the Ang II type I receptors) suppresses the impaired effects in the rat hippocampus. The expression of Ang II is high in the hippocampus, therefore its role in the processing of cognitive functions, such as the hippocampus is undeniable. Furthermore, the recent research on the role of AT2 receptors in cognitive processes provided evidence about their positive effect on cognitive processes (9).

An interesting area within the context of stress-pain interactions is the relationship between chronic pain and affective disorders. As mentioned before exposure to stressful conditions, results in SIH (10). Following the effect of losartan treatment on physical and psychological stressors, it significantly changed the pain threshold in the tail flick test. Also, in agreement with current findings, some investigations showed the same results that losartan diminished pain (15, 16). Thus, in the present study, we determined that the administration of losartan may produce an antinociceptive effect. 

## Conclusion

It seems losartan has positive impacts on anxiety, memory, and pain. Although further studies are needed to determine whether using a different dosage of losartan leads to the same outcomes. 
